# Fibroblast-myofibroblast transition is differentially regulated by bronchial epithelial cells from asthmatic children

**DOI:** 10.1186/s12931-015-0185-7

**Published:** 2015-02-13

**Authors:** Stephen R Reeves, Tessa Kolstad, Tin-Yu Lien, Sarah Herrington-Shaner, Jason S Debley

**Affiliations:** Division of Pulmonary Medicine, MS OC.7.20, Seattle Children’s Hospital, 4800 Sand Point Way NE, PO Box 5371, Seattle, WA 98105 USA; Center for Immunity and Immunotherapies, Jack R. MacDonald Building, Seattle Children’s Research Institute, 1900 9th Ave, Seattle, WA 98109 USA; Department of Pediatrics, University of Washington School of Medicine, Seattle, USA

**Keywords:** Air-liquid interface culture, Airway remodeling, Asthma, Bronchial epithelial cells, Cell culture, Fibroblasts, Myofibroblasts, α-smooth muscle actin, TGFβ_2_

## Abstract

**Background:**

Airway remodeling is a proposed mechanism that underlies the persistent loss of lung function associated with childhood asthma. Previous studies have demonstrated that human lung fibroblasts (HLFs) co-cultured with primary human bronchial epithelial cells (BECs) from asthmatic children exhibit greater expression of extracellular matrix (ECM) components compared to co-culture with BECs derived from healthy children. Myofibroblasts represent a population of differentiated fibroblasts that have greater synthetic activity. We hypothesized co-culture with asthmatic BECs would lead to greater fibroblast to myofibroblast transition (FMT) compared to co-culture with healthy BECs.

**Methods:**

BECs were obtained from well-characterized asthmatic and healthy children and were proliferated and differentiated at an air-liquid interface (ALI). BEC-ALI cultures were co-cultured with HLFs for 96 hours. RT-PCR was performed in HLFs for alpha smooth muscle actin (α-SMA) and flow cytometry was used to assay for α-SMA antibody labeling of HLFs. RT-PCR was also preformed for the expression of tropomyosin-I as an additional marker of myofibroblast phenotype. In separate experiments, we investigated the role of TGFβ_2_ in BEC-HLF co-cultures using monoclonal antibody inhibition.

**Results:**

Expression of α-SMA by HLFs alone was greater than by HLFs co-cultured with healthy BECs, but not different than α-SMA expression by HLFs co-cultured with asthmatic BECs. Flow cytometry also revealed significantly less α-SMA expression by healthy co-co-cultures compared to asthmatic co-cultures or HLF alone. Monoclonal antibody inhibition of TGFβ_2_ led to similar expression of α-SMA between healthy and asthmatic BEC-HLF co-cultures. Expression of topomyosin-I was also significantly increased in HLF co-cultured with asthmatic BECs compared to healthy BEC-HLF co-cultures or HLF cultured alone.

**Conclusion:**

These findings suggest dysregulation of FMT in HLF co-cultured with asthmatic as compared to healthy BECs. Our results suggest TGFβ_2_ may be involved in the differential regulation of FMT by asthmatic BECs. These findings further illustrate the importance of BEC-HLF cross-talk in asthmatic airway remodeling.

## Background

Asthma is the most common chronic disease in children, affecting at least 6 million children and causing greater than 7 million physician visits and 200,000 hospitalizations annually in the United States alone [1]. In recent years, our understanding of the pathogenesis of persistent bronchial asthma has advanced significantly. We now appreciate that the obstructive lung disease incurred as a result of asthma occurs during childhood [2] and does not improve as a child ages into adulthood [3] or with treatment using current asthma therapies [4-6]. Contemporary views of asthma pathophysiology now include disordered wound repair and associated airway remodeling as chief components underlying the obstructive lung disease that is associated with asthma in addition to chronic inflammatory changes [7]. Furthermore, we now understand that the airway epithelium undergoes significant structural changes early in asthma. Given that it is the first point of contact between the host airways and the environment, a new paradigm of asthma pathogenesis has emerged to potentially explain asthma progression and airway remodeling, wherein ongoing injury, irritation, and/or viral infection of airway epithelial cells results in disordered wound repair, including disordered epithelial modulation of lung fibroblast and airway smooth muscle activity [8].

Multiple previous studies have identified sub epithelial thickening and deposition of extra cellular matrix (ECM) as common features of airway biopsies from both adults and children [9,10]. Recent data from *in vitro* studies in our laboratory has demonstrated that human lung fibroblasts (HLFs) co-cultured with primary human bronchial epithelial cells (BECs) produce greater quantities of ECM components when the co-cultures were performed with BECs obtained from asthmatic children, suggesting altered regulation of the epithelial-fibroblast cross-talk [11]. While lung fibroblasts have been extensively studied in the context of pulmonary fibrosis [12], their inherent role in asthmatic airway remodeling is just beginning to be elucidated [13,14]. Previous studies have demonstrated that mesenchymal cells such as fibroblasts are the predominant source of many ECM proteins. The latter is particularly true for fibroblasts that have differentiated into a myofibroblast phenotype [15,16], i.e. alpha smooth muscle actin (α-SMA) expressing fibroblasts. Myofibroblasts are known to be the primary source of type I and III collagen in fibrotic lesions and this is a consequence of a phenotype differentiation that is dependent on stimulation by transforming growth factor beta (TGFβ) [17-19]. Furthermore, myofibroblasts represent a contractile phenotype that may also directly participate in scar formation and contraction and this may be enhanced in primary bronchial fibroblasts derived from asthmatics [20]. BECs have been demonstrated to influence fibroblast-to-myofibroblast transition (FMT), possibly via paracrine signaling [21]; however, the role of primary BECs derived from asthmatic children versus those obtained from healthy children in FMT has not been described.

In order to further investigate the role that BECs play in the regulation of FMT, we employed a co-culture model to evaluate the differences in HLF cultures that were either co-cultured with healthy or asthmatic BECs. We hypothesized that the HLFs co-cultured with primary BECs derived from asthmatic children would display a greater degree of FMT compared to HLFs co-cultured with BECs derived from healthy children. Furthermore, the presence of greater numbers of myofibroblasts could account for the enhanced deposition of ECM that has been previously described in our model system and may further explain the degree of airway remodeling that has been reported in asthmatic children.

## Methods

### Subjects

Atopic asthmatic and healthy non-atopic non-asthmatic children ages 6–18 years who were undergoing an elective surgical procedure requiring endotracheal intubation and general anesthesia were recruited for this study. A detailed medical history was obtained at enrollment to ensure that participants met the following inclusion and exclusion criteria. Children with asthma had at least a 1 year history of physician-diagnosed asthma, physician documented wheezing in the 12 months prior to study enrollment, used a short-acting beta-agonist (albuterol) ≥ twice a month or were taking a daily inhaled corticosteroid or leukotriene receptor antagonist, and were born at ≥ 36 weeks gestation. Healthy subjects were born at ≥ 36 weeks gestation, had no history of asthma, reactive airway disease, chronic daily cough, or physician-diagnosed obstructive lung disease, and no history of prior treatment with a systemic or inhaled corticosteroid, short-acting beta-agonist (albuterol), or oxygen. Children with asthma had one or more of the following atopic features: history of a positive skin prick test or positive radioallergosorbent testing (RAST) for a common aeroallergen (discussed below), elevated serum IgE (>100 IU/mL), history of physician-diagnosed and treated allergic rhinitis, history of physician-diagnosed and treated atopic dermatitis. Healthy subjects lacked a history of any of the above atopic features and were excluded if they had any other atopic comorbidity.

From each subject, a blood sample was drawn and used to measure total serum IgE and RAST allergen-specific IgE to dust mites (*D. farina and D. pteronyssinus*), cat epithelium, dog epithelium, *alternaria tenuis, aspergillus fumigatus*, and timothy grass. The fraction of exhaled nitric oxide (FE_NO_) was measured according to American Thoracic Society (ATS) guidelines using a NIOX MINO nitric oxide analyzer (Aerocrine®, Sweden) [22]. Forced vital capacity (FVC), forced expiratory volume in 1 second (FEV_1_), and forced expiratory flow between 25% and 75% of FVC (FEF_25–75_) were measured according to ATS guidelines using a VMAX® series 2130 spirometer (VIASYS Healthcare, Hong Kong). Spirometry was repeated 15 minutes following administration of 2 puffs of albuterol in children with asthma.

Written consent was obtained from parents of subjects and assent was obtained for children ≥ age 7 years. The Seattle Children’s Hospital Institutional Review Board approved this study.

### Epithelial cell isolation, proliferation, and differentiation

Immediately after the endotracheal tube was secured three bronchial epithelial cell samples were obtained from subjects while under general anesthesia using 4 mm Harrell® unsheathed bronchoscope cytology brushes (CONMED® Corporation). As described by Lane et al. [23], the unprotected brush was inserted through an endotracheal tube, advanced until resistance was felt, and rubbed against the airway surface for 2 seconds. Cells were seeded onto T-25 cell culture flasks pre-coated with type I collagen and proliferated under submerged culture conditions. Using passage 2 cells, epithelial cells were differentiated at an air-liquid interface (ALI) using methods previously described by our lab [24].

### BEC-fibroblast co-cultures

HLFs from a healthy child were obtained from a commercial vender (Lonza, Walkersville, MD) and the same passage of HLFs were used for all co-culture experiments. HLF cultures were established using a Fibroblast Cell Media BulletKit™ (FGM) per Lonza recommendations. HLF were seeded at a density of ~2,500 cells/cm^2^ in 12-well Collagen I BD BioCoat™ plates (Becton Dickinson, Bedford, MA) and incubated for 7 days to achieve a confluent monolayer prior to initiation of BEC-HLF co-cultures. Media changes of FGM occurred at 48 hour intervals. At 48 hours prior to experimental Day 0, the HLF media was replaced with co-culture media (1:1 FGM and PneumaCult ALI Maintenance Media). ALI transwells were placed in co-culture with the HLF cells at experimental Day 0 by transferring the transwell inserts to the well plates containing the established HLF cultures. The transwells inserts containing the BEC ALI culture were in close proximity to the submerged HLF cultures contained in the basolateral chamber (~1 mm between the well plate and the permeable transwell bottom) and shared the same media. The co-culture media in the basolateral chamber was changed every 24 hours and stored at −80°C for subsequent analysis. The media and cells were collected for studies 96 hours following initiation of co-culture experiments.

### RNA extraction and real-time PCR

Total RNA was isolated from HLF cells co-cultured with BEC’s grown at an ALI. Three wells from each experimental condition were harvested and pooled to isolate RNA using the RNAqueous kit for total RNA purification from Ambion®-Applied Biosystems (Austin, TX). RNA concentration and integrity were determined using the Agilent® 2100 Bioanalyzer system and Agilent® RNA 6000 Nano Chips (Agilent® Technologies, Foster City, CA). RNA samples (1 μg) with a RNA integrity number (RIN) ≥ 8 were reverse transcribed with MMLV reverse transcriptase with a combination of random hexamers and oligo-dTs using the SuperScript® VILO cDNA Synthesis Kit (Life Technologies, Grand Island, NY). Samples were diluted up to a final volume of 100 μl (10 ng/μl). Semi-quantitative real-time qPCR was performed using validated TaqMan® probes (Life Technologies, Grand Island, NY) for alpha smooth muscle actin (α-SMA). Assays were performed using the TaqMan® Fast Advanced Master Mix reagents and accompanying protocol and the Applied Biosystems StepOnePlus™ Real-Time PCR System with StepOne Software v2.2.2 (Life Technologies, Grand Island, NY).

### Immunohistochemistry (IHC)

Sterilized 12 mm round glass coverslips were coated with type I collagen and placed in the bottom of one of the replicate chambers of the 12 well plates prior to seeding the HLFs. Following 96 hrs of co-culture, the coverslips were carefully removed and placed in a separate 12 well plate for IHC. Coverslips containing cells were then washed 3 times in room temperature PBS to remove residual media and were then fixed with 50:50 methanol and acetone at 20°C for 10 minutes. Coverslips were then washed with PBS and blocked with 10% FBS for 30 minutes. Following this, coverslips were washed again with PBS and then incubated with an α-SMA-FITC primary antibody (clone 1A4, 1:500; Sigma Aldrich, St Louis, MO). Coverslips were then washed in PBS three times and were then mounted on to slides using ProLong® Gold antifade reagent with DAPI (Life Technologies, Grand Island, NY). Images were acquired using an automated Leica DM6000B fluorescent microscope (Leica Microsystems, Wetzlar, Germany) in 7 × 7 grids at 400 × and stitched together using LASAF software to provide higher resolution images of larger areas of tissue.

In order to enumerate the number of cells present per field, anaylsis of DAPI stained nuclei was performed in addition to α-SMA staining described above. Stitched images were inverted, cropped to identical pixel densities (9000 × 6000), and saved as binary image files (Adobe Photoshop CS 5.1, San Jose, CA). Images were then imported into ImageJ (National Institutes of Health, Bethesda, MD) where the images were processed using the automatic threshold setting to mitigate any sampling bias. Positive stained nuclei were counted using the particle analysis feature.

### Flow cytometry

Following the 96 hour co-culture exposure HLFs from each experimental group were mechanically detached from the culture wells and suspended in PBS. Samples were snap frozen in liquid nitrogen so that samples from each group were run in parallel. Before the flow cytometry protocol began, the cells were quickly thawed and then centrifuged for 5 minutes at 200 × g and suspended in PBS for two washings. The cells were then fixed and permeabilized in ice cold 50:50 methanol/acetone for 10 minutes. Subsequently the cells were washed twice with Flow Cytometry Staining Buffer (Cat. 00–4222, eBioscience, San Diego, CA). After these washes the HLF were incubated with anti-α-SMA conjugated to FITC (clone 1A4, 1:1000; Sigma Aldrich, St Louis, MO) for 45 minutes at room temperature negative controls were maintained in parallel and were not stained in this step. Cells were then twice washed again with Flow Cytometry Staining Buffer and the data was then acquired with a LSR II flow cytometer (Becton Dickinson, Bedford, MA). Both negative controls (unstained HLF) and positive controls (>2 week old HLF cultures) were used to set gating parameters for data acquisition. Data was analyzed following acquisition with FlowJo 8 (Treestar INC, Ashland, OR).

### Monoclonal antibody inhibition studies

HLF cultures at 7 days were exposed to TGFβ_2_ (1 μg/mL, R&D Systems, Minneapolis, MN), monoclonal neutralizing antibody (Mab) to TGFβ_2_ (10 μg/mL, Sigma Aldrich, St Louis, MO), and/or PGE_2_ (40 μg/mL, Cayman Chemical Company, Ann Arbor, MI) for 48 hrs. Given the short half-life of PGE_2_, media was refreshed with 40 μg/mL every 6 hours during experiments. RNA was extracted after 48 hours of exposure and expression of α-SMA was assessed using qPCR. In separate experiments, HLF co-cultured with healthy or asthmatic BEC cultures were exposed every 24 hours to TGFβ_2_ Mab (10 μg/mL, Sigma Aldrich, St Louis, MO) for 96 hours, at which time RNA was extracted and expression of α-SMA was assessed using qPCR.

Gene expression was normalized to GAPDH in all experiments.

### Statistical analysis

For clinical parameters including age, lung function and FE_NO_, the paired t-test was used for comparisons between asthmatic and healthy subjects as data was normally distributed within each subject group. The Wilcoxon signed-rank test was used to compare gender and IgE levels because the distributions among asthmatic subjects were non-normally distributed. For RT-PCR studies, the relative expression of α-SMA was normalized using glyceraldehyde 3-phosphate dehydrogenase (GAPDH) as a non-regulated reference gene. Analyses of real-time qPCR results were performed using GenEx version 5.0.1 (MultiD Analyses AB, Göteborg, Sweden) based on methods described by Pfaffl [25]. Statistical significance was set at *p* < 0.05. For flow cytometry data, pairwise comparisons across the groups were used for non-parametric data (Mann Whitney U) with a significance level set at p < 0.05. For cell enumeration data, the Kruskal–Wallis test was used to make comparisons among the groups, as this data was not normally distributed. Statistical analyses of clinical data, flow cytometry data, and cell enumeration data were performed using Prism® 6.0 software (GraphPad Software Inc., San Diego, CA).

## Results

Lower airway cells were obtained by bronchial brushings from 10 healthy and 10 asthmatic children as defined by the inclusion criteria discussed in the methods section. Primary BEC cultures were derived from these samples and used for all portions of the experiment. Summary data for the patient characteristics is displayed in Table 1. The ages of the subjects were comparable (healthy 10.3 +/− 3.6 vs. asthmatic 11.7 +/− 3.4; p = 0.4); however, our asthmatic group contained a greater number of males. The majority of our asthmatic subjects were using daily inhaled corticosteroids (ICS; 90%). Additionally, most of the asthmatic subjects had positive RAST testing to a specific aeroallergen (90%). Asthmatic subjects also displayed a significantly higher serum IgE compared to healthy subjects (916 IU/mL in asthmatic subjects vs 21.3 IU/mL in healthy subjects; p < 0.0005). While most parameters of the pulmonary function tests were comparable between the groups including FE_NO_ levels (14.9 +/− 8.8 vs. 9.3 +/− 4.6; p = 0.2), the FEV_1_/FVC ratio was significantly lower in asthmatic as compared to healthy subjects (83% +/− 5% vs. 89% +/− 4%, respectively; p = 0.04). In all, these findings are representative of the patient population seen at Seattle Children’s Hospital and represent an asthmatic population with fairly mild disease.

**Table 1:** **Characterization of subjects**

	**Healthy Controls**	**Asthmatics**	**P value**
	**(N = 10)**	**(N = 10)**	
Age yrs. (SD)	10.3 (3.6)	11.7 (3.4)	0.4
Females (%)	60%	20%	0.07
History of Eczema (yes; %)	0	4 (40%)	N/A
History of Allergic Rhinitis (yes; %)	0	9 (90%)	N/A
Positive RAST (yes; %)	0	9 (90%)	N/A
Use of inhaled steroids at enrollment (yes; %)	0	9 (90%)	N/A
IgE IU/mL (SD)	21.3 (12.4)	916 (1616)	0.0005
FVC % predicted (SD)	101 (13.2)	100.7 (11.5)	0.9
FEV_1_/FVC Ratio (SD)	0.89 (0.04)	0.83 (0.05)	0.04
FEV_1_ % predicted (SD)	99.8 (14.5)	96.6 (12.1)	0.6
FEF_25–75_ % predicted (SD)	99 (20.4)	88 (18.5)	0.2
FENO ppb (SD)	9.3 (4.6)	14.9 (8.8)	0.2

RAST = radioallergosorbent testing; FVC = forced vital capacity; FEV1 = forced expiratory volume in 1 second; FEF25-75 = forced expiratory flow between 25% and 75% of expiration; SD = standard deviation.

**Figure 1:**
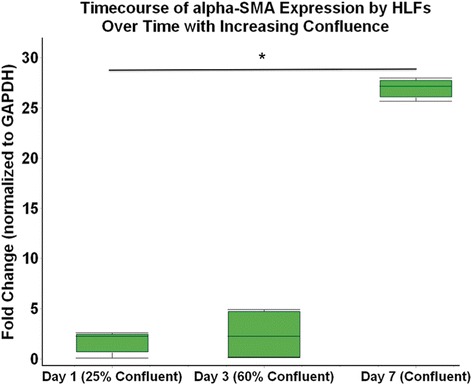
**Time course of α-SMA expression in HLF cultures.** The expression of α-SMA by HLF cultures is shown following initial seeding through Day 7 (start of co-culture exposures). Expression of α-SMA at Day 7 of culture was 25 fold higher than Day 1 (p < 0.0001).

**Figure 2:**
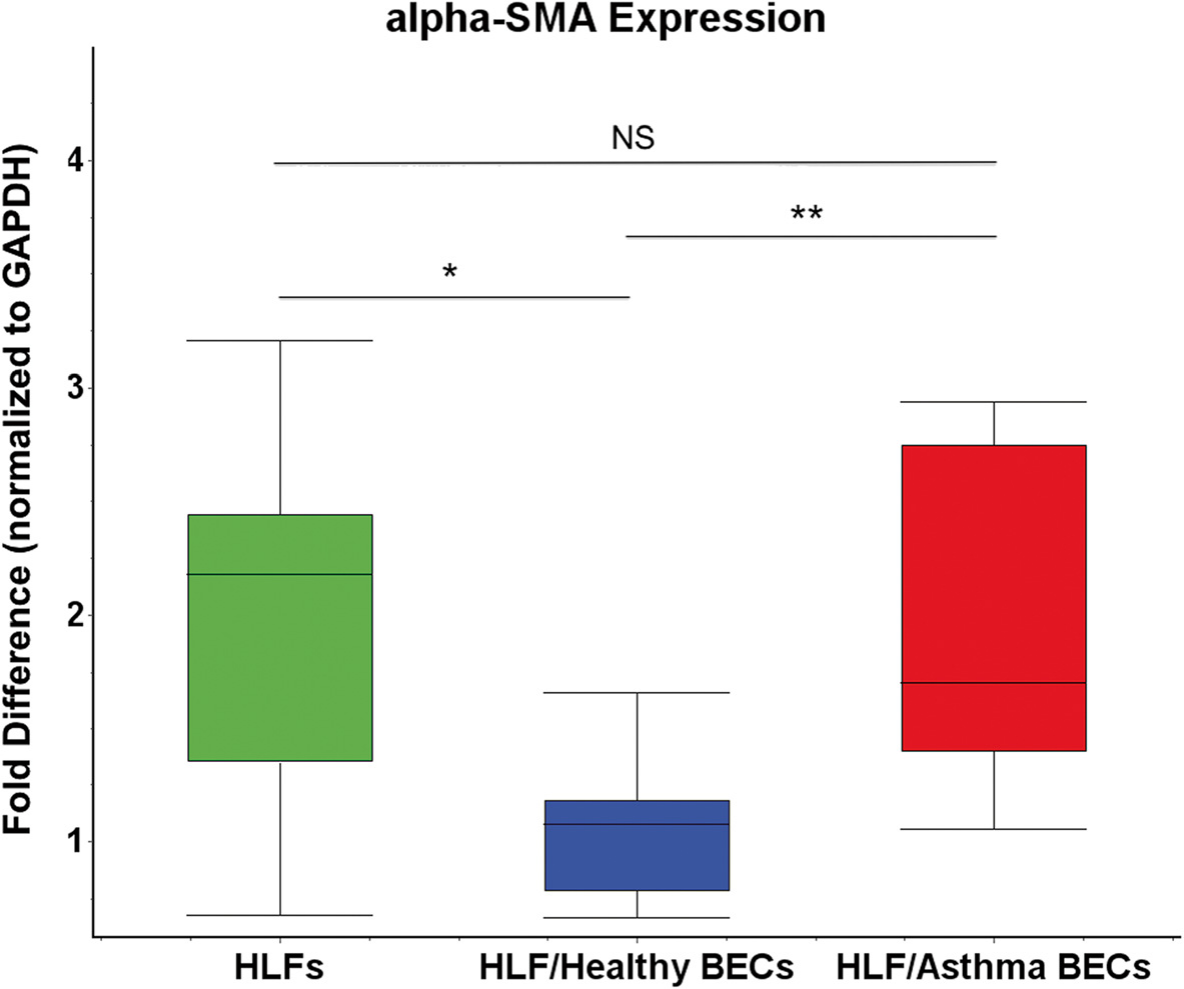
**Expression of α-SMA mRNA following 96 hours of co-culture.** Expression of α-SMA by HLFs cultured alone was 1.9 fold higher than by HLFs co-cultured with healthy BECs (p = 0.008; *). α-SMA expression by HLFs co-cultured with asthmatic BECs was 2-fold higher than by HLFs co-cultured with healthy BECs (p = 0.002; **). There was no difference in α-SMA expression between HLFs cultured alone and HLFs co-cultured with asthmatic BECs.

In order to characterize the trajectory of endogenous FMT within our HLF cell lines, mRNA was isolated along a time course study following seeding at day 1 (~25% confluent), day 3 (~60% confluent), and day 7 (100% confluent). Expression of α-SMA was used as a surrogate for FMT and at day 7 following seeding was significantly greater than at day 1 (25-fold increase, p < 0.0001; Figure 1). Next, expression of α-SMA mRNA was examined following 96 hours of co-culture with either BECs derived from healthy children or BECs derived from asthmatic children (Figure 2). Time controls of HLF alone were maintained in parallel and are shown for comparison. Expression of α-SMA mRNA by HLFs co-cultured with healthy BEC was 1.9 fold lower than HLF alone (p = 0.008). In contrast, HLFs co-cultured with asthmatic BECs displayed no significant difference when compare to HLFs alone; however, the asthmatic BEC-HLF co-cultures expressed 2-fold greater α-SMA compared to healthy BEC-HLF co-cultures (p = 0.002).

**Figure 3:**
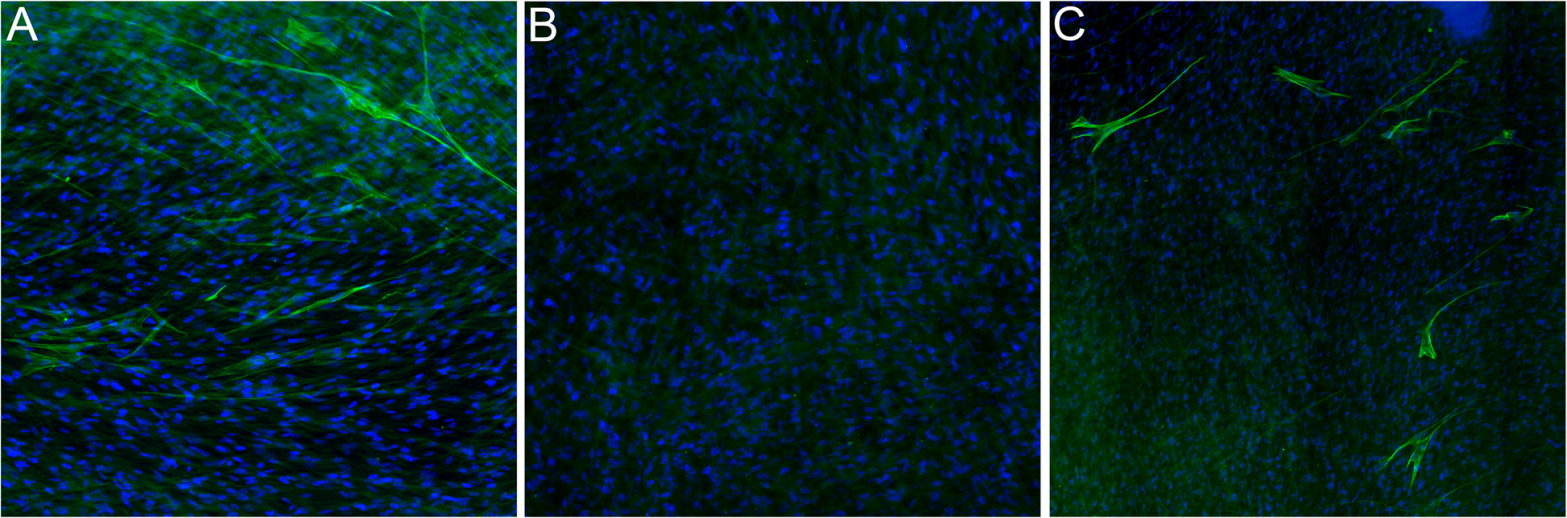
**Immunostaining for α-SMA in HLF alone or co-cultured with healthy or asthmatic BECs.** Immunostaining for α-SMA in HLF Alone **(Panel A)**, HLF Co-Cultured with heathy BECs **(Panel B)**, and HLF Co-Cultured with asthmatic BECs **(Panel C)** following 96 hr exposure. All sections are counterstained for DAPI.

**Figure 4:**
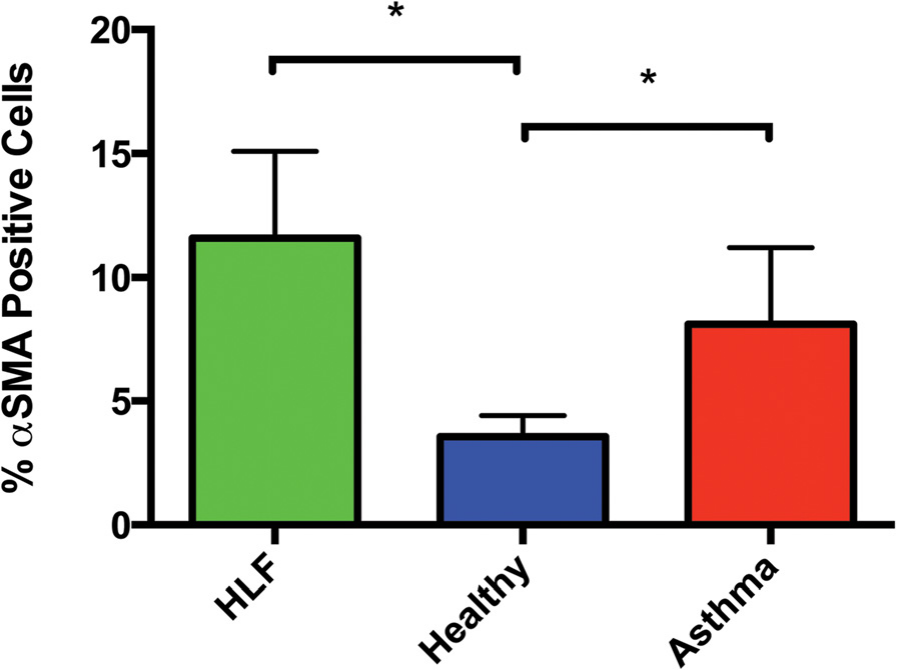
**Quantification of α-SMA positive cells by flow cytometry.** Flow cytometry for α-SMA (% positive) in HLF Alone, HLF Co-Cultured with Heathy BECs, and HLF Co-Cultured with Heathy BECs following 96 hr exposure. Flow cytometry revealed 3.6% α-SMA positive cells in the healthy co-culture group compared to 8.1% α-SMA positive cells in the asthmatic co-culture group (p < 0.03) and 11.6% α-SMA positive cells in the HLF alone (p < 0.03).

Immunostaining of the HLF for α-SMA revealed qualitatively greater staining in the HLF alone group compared to either the HLF co-cultured with healthy BEC or asthmatic BEC. However, considerably greater staining was observed in the HLF co-cultured with BEC derived from asthmatic children (representative images are shown in Figure 3). In order to quantify the immunostaining of the HLF for α-SMA, the same antibody that was used for immunohistochemistry was also used for flow cytometry experiments in a subset of samples (n = 4 per group). Flow cytometry staining for α-SMA in HLF cells alone following the 96-hour experiment revealed 11.6% positively stained cells, which significantly was greater than HLF co-cultured with healthy BEC (3.6%; p < 0.03; Figure 4). Staining for α-SMA positive HLF was greater in HLF co-cultured with asthmatic BEC compared to healthy BEC (p < 0.03). No significant differences emerged between HLF cultured alone and HLF co-cultured with asthmatic BEC.

**Figure 5:**
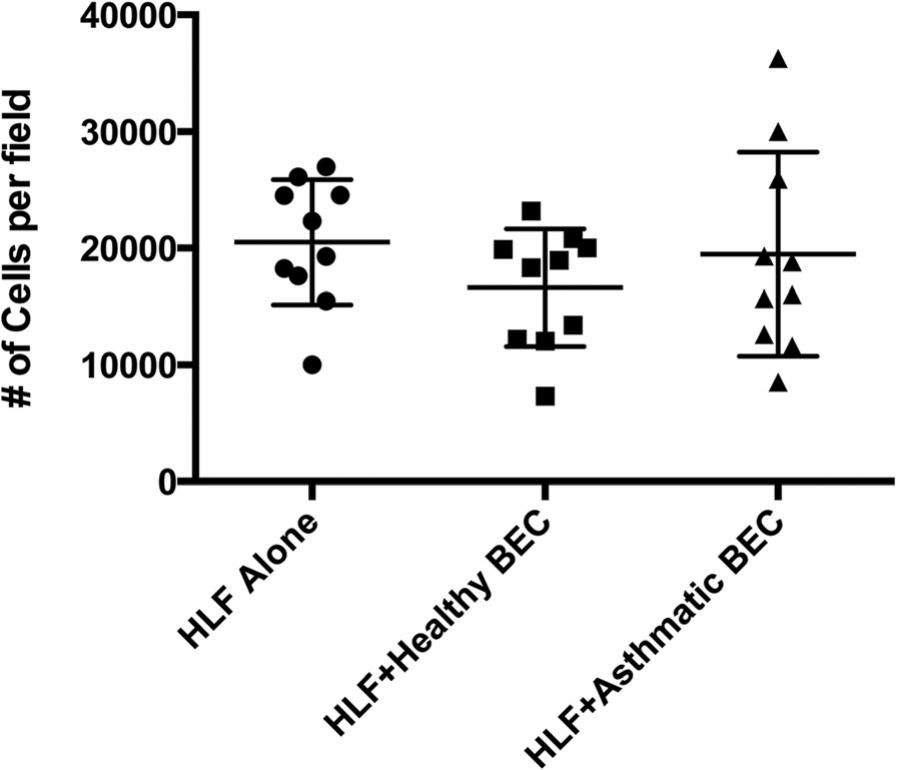
**Quantification of total number of HLF cells in HLF cultured alone compared to co-culture with healthy or asthmatic BECs.** Cell counts as demonstrated by software enumeration of DAPI positive nuclei. No significant differences were observed among the three HLF groups (n = 10 per group).

In order to ascertain whether the findings of differential α-SMA staining in the above experiments was related to differing cell densities present in the HLF cell cultures versus an alteration in the overall expression of α-SMA, non-bias computer automated counts of DAPI stained nuclei were also evaluated in the immunohistochemistry sections (n = 10 per group). No significant differences in the number of DAPI stained nuclei were observed among the groups (Figure 5).

**Figure 6:**
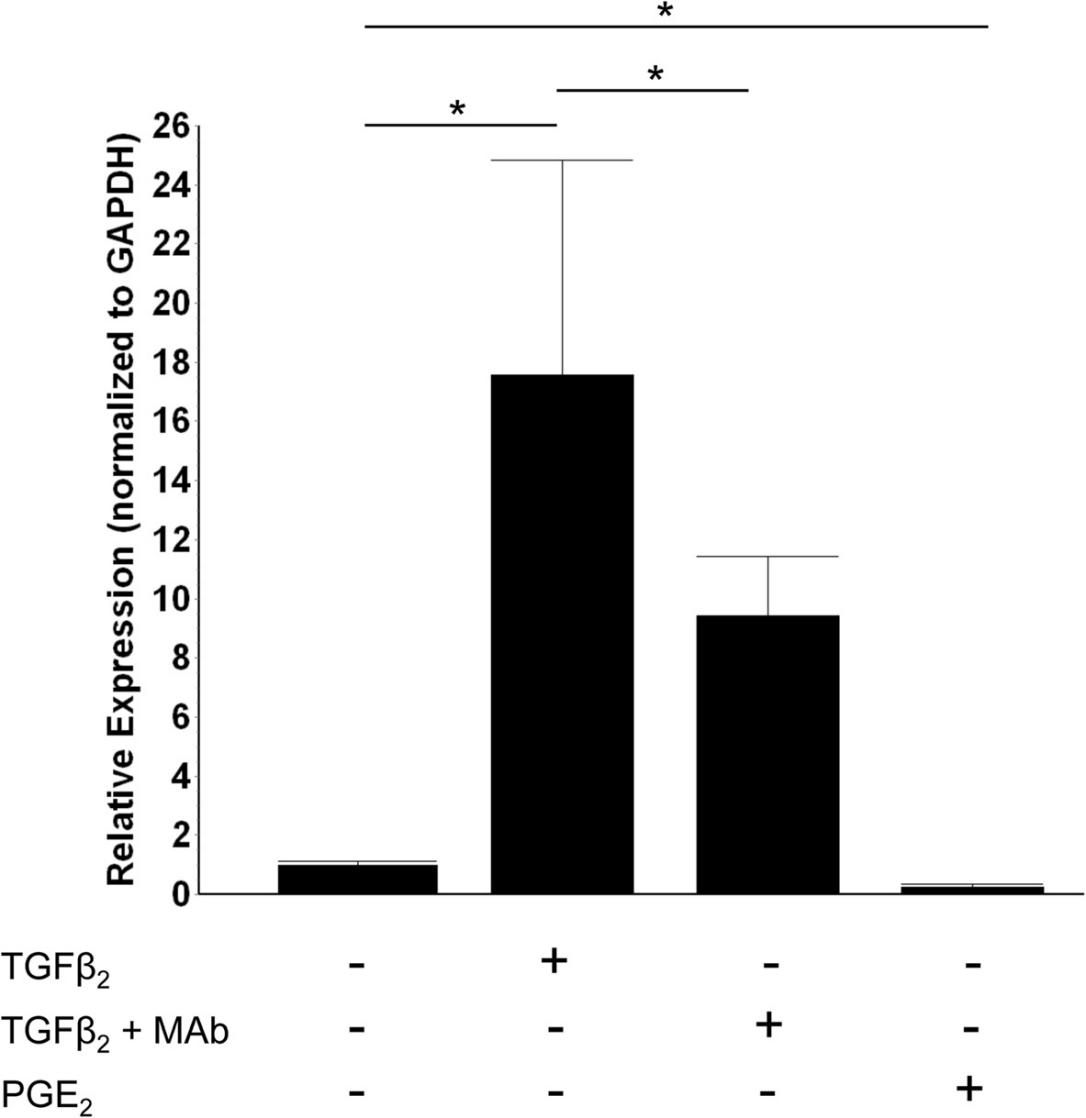
**Expression of α-SMA mRNA in HLF following exposure to TGFβ**_**2,**_ **TGFβ**_**2**_ **monoclonal neutralizing antibody, and PGE**_**2.**_ **.** TGFβ_2_ exposure lead to a 17-fold increase in α-SMA expression (p < 0.001). This effect was decreased by nearly 50% with addition of TGFβ_2_ neutralizing antibody (p < 0.01). Addition of PGE_2_ lead to an 80% reduction in α-SMA expression compared to untreated HLF (p < 0.01).

In our previous studies, we have demonstrated greater expression of active TGFβ_2_ in asthmatic BEC-HLF co-cultures despite no significant differences in the expression of active TGFβ_1_ when compared to either healthy BEC-HLF co-cultures or HLF alone [11]. To further investigate the effects of TGFβ_2_ signaling in the present study, HLF cultures were exposed to exogenous TGFβ_2_ with and without the addition of monoclonal anti-TGFβ_2_ antibody (n = 6 per group). Administration of exogenous TGFβ_2_ lead to a 17-fold increase in α-SMA expression (p < 0.001; Figure 6). The addition of monoclonal antibody to TGFβ_2_ ameliorated the TGFβ_2_-mediated increase in α-SMA expression by about 50% (p < 0.01). The addition of exogenous PGE_2_ led to an 80% reduction in α-SMA expression compared to untreated HLFs (p < 0.01).

**Figure 7:**
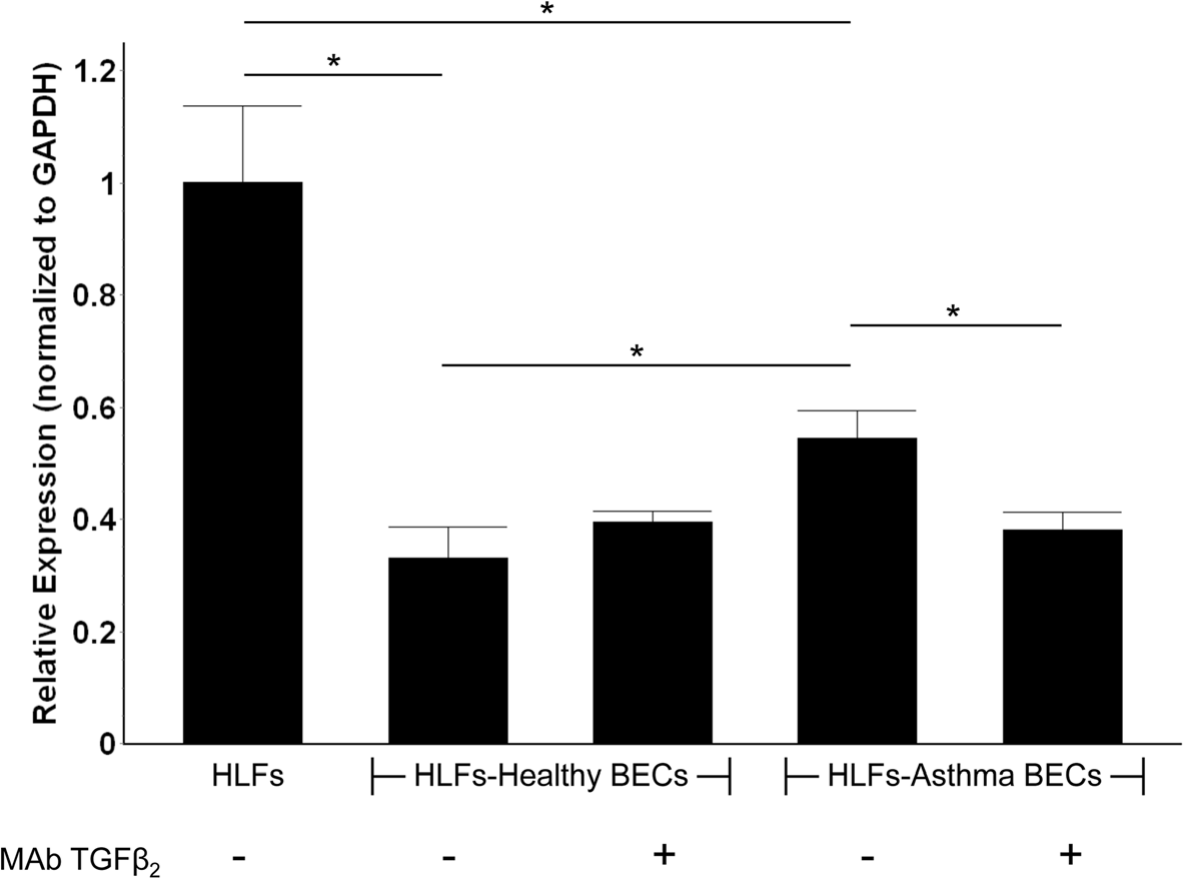
**Expression of α-SMA in HLF co-cultured with either healthy BEC or asthmatic BEC in the presence of TGFβ**_**2**_ **monoclonal neutralizing antibody.** Expression of α-SMA by HLF was greater than both HLF-healthy BEC and HLF asthmatic BEC co-cultures. HLF-asthmatic BEC co-cultures demonstrated 40% greater expression of α-SMA compared to HLF-healthy BEC co-cultures (p < 0.01). Addition of TGFβ_2_ neutralizing antibody did not significantly change α-SMA expression in HLF-healthy BEC co-cultures; however, similar treatment lead to a 30% reduction in the expression of α-SMA in HLF-asthmatic co-cultures (p < 0.01).

Furthermore, we then conducted experiments using a subset of asthmatic and healthy BECs (n = 3 per group; Figure 7) to test the effect of the addition of TGFβ_2_ neutralizing monoclonal antibody to HLF co-cultured with BECs. Similar to experiments outlined in Figure 2, expression of α-SMA by HLFs co-cultured with healthy and asthmatic BECs was significantly less than by HLFs alone. Expression of α-SMA by HLFs co-cultured with healthy BECs was 40% lower than by HLFs co-cultured with asthmatic BECs (p < 0.01). The addition of TGFβ_2_ neutralizing monoclonal antibody to healthy BEC-HLF co-cultures did not significantly change expression of α-SMA by HLFs, whereas the addition of TGFβ2 neutralizing monoclonal antibody to asthmatic BEC-HLF co-cultures led to a 30% reduction in expression of α-SMA by HLFs (p < 0.01).

**Figure 8:**
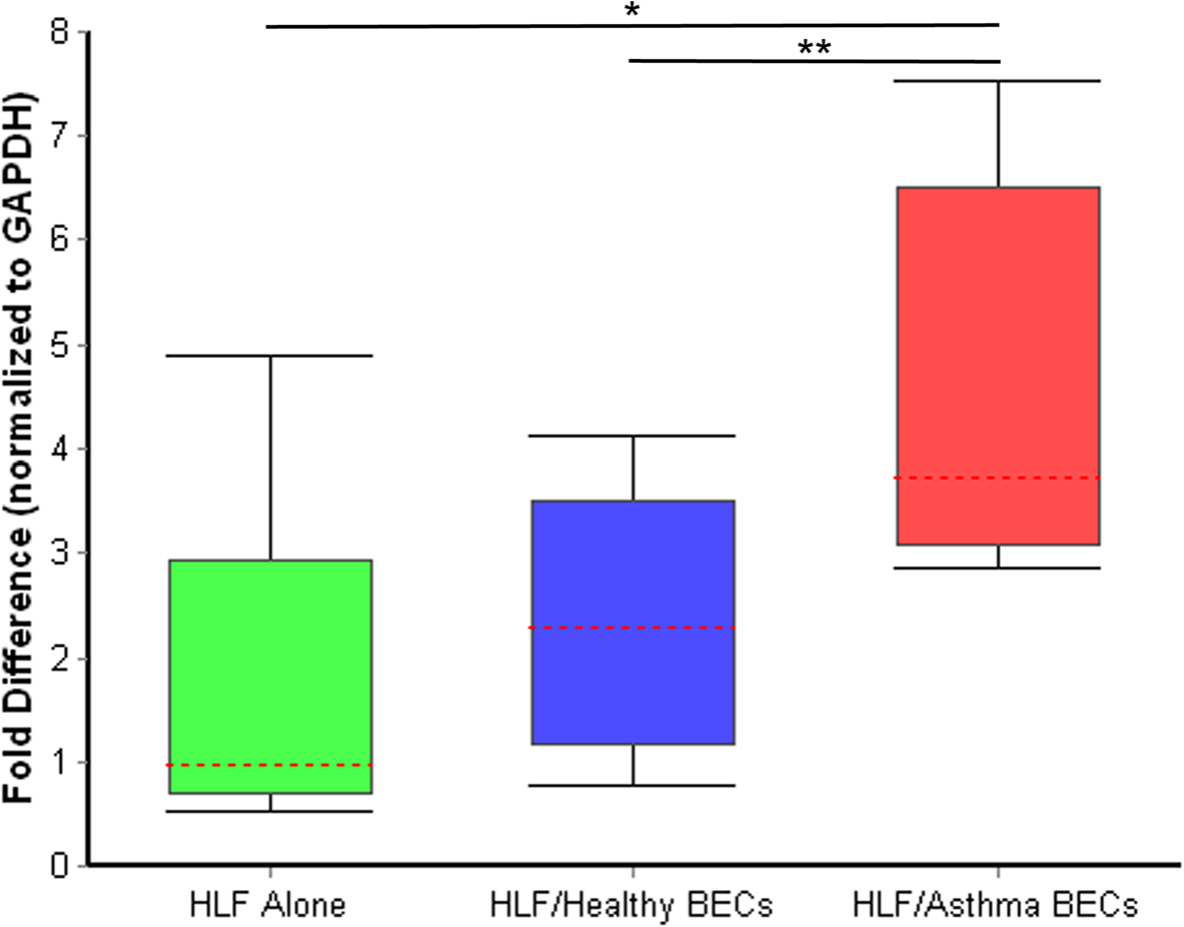
**Expression of tropomyosin-I following 96 hours of co-culture.** Expression of tropomyosin-I by HLFs cultured alone was not significantly different than by HLFs co-cultured with healthy BECs. Tropomyosin expression by HLFs co-cultured with asthmatic BECs was 4.3-fold higher than by HLFs co-cultured with healthy BECs (p = 0.03) and 6.2-fold greater than HLF alone (p < 0.004).

In addition to the experiments conducted above involving the examination of α-SMA expression, we also examined the expression of tropomyosin-I as a second marker of the myofibroblast phenotype (Figure 8). Interestingly we did not observe an inherent up regulation of tropomyosin-I in the HLF cultured alone. Expression of tropomyosin-I was not significantly different between HLF alone and HLF co-cultured with healthy BEC co-cultures following 96 hours of co-culture. In contrast, expression of tropomyosin-I in HLF co-cultured with asthmatic BEC co-cultures was greater than HLF alone or HLF co-cultured with healthy BEC (6.2 fold p < 0.004; 4.3 fold p < 0.03, respectively).

## Discussion

In this study, we demonstrate for the first time that co-culture of HLFs with BECs derived from a healthy pediatric donor significantly down-regulates FMT in cultured HLF cells. Furthermore, we demonstrate that co-culture of the same HLF cell line with BECs derived from asthmatic children does not produce the same degree of FMT down-regulation. Conversely, these co-cultured HLF have a comparable degree of FMT to HLF cells cultured alone suggesting impaired regulatory signaling from the asthmatic derived BECs. Differences observed among the three HLF groups could not be accounted for by alterations in the cell density suggesting that the increased expression of α-SMA as a marker of FMT was related to differential gene expression and not altered cellular proliferation in the established HLF cultures. These findings lend further support to concept that the epithelial cells of the airway exert regulatory influence on the other cell types of the airway. The findings are significant in the context of asthmatic airway remodeling and may provide further insight into the pathophysiology behind the increased airflow obstruction observed in this population. Furthermore, this study adds additional evidence to the paradigm that airway epithelium influences the behavior of other cells types in the epithelial-mesenchymal unit.

A consistent finding in both studies of adult and pediatric asthma is the occurrence of sub-epithelial fibrosis and deposition of ECM [9,10]. The matrix deposition that occurs in these subjects is largely comprised of collagens, fibronectin and other ECM constituents that are deposited by mesenchymal cells such as fibroblasts. Fibroblasts may arise from multiple different lineages (reviewed in Hu and Phan 2013, [26]); however, they are normally present within multiple tissues including lung and respond to a variety of signals associated with tissue damage to proliferate and undergo FMT. In contrast, myofibroblasts are not typically found in healthy tissue, but arise in response to signaling associated for tissue damage and wound repair. Myofibroblasts are identifiable from resident fibroblasts by the expression of α-SMA and are known to be a predominant source of type I collagen and respond to multiple pro-fibrotic and inflammatory cytokines including TGFβ [27,28]. The role of TGFβ in driving FMT is well established and has been tied to the enhanced production of collagen associated with the acquisition of the myofibroblast phenotype [18]. Regulation of the transition to a myofibroblast phenotype is complex and has been shown to involve several signaling pathways including Wnt, Notch, and Hedgehog in addition to TGFβ and associated downstream mediators of these respective pathways [26]. Suppression of α-SMA gene expression has been shown to be associated with decreased expression of collagen production establishing a direct link between the myofibroblast phenotype and collagen production [29]. While the presence of myofibroblasts is essential for the deposition of ECM and wound contracture in normal wound healing, over activation can lead to disordered wound repair, hypertrophic scarring, and fibrosis [27,30].

Holgate and others have proposed that the airway epithelium is central to asthma pathogenesis and may also be a driving force behind that the airway remodeling that is incurred by asthmatic subjects [7]. In this paradigm, epithelial cells sustain repeated insults through interaction with environmental factors such viruses, dust mites, pollen, and other infections that result in recruitment of inflammatory cells leading a chronic wound state. The latter process invokes endogenous repair mechanisms through the production of cytokines and growth factors that orchestrate wound repair via interactions with other cell types such as fibroblasts. However, in the case of asthma, the repair of the epithelium is hypothesized to be incomplete or dysregulated, resulting in the loss of epithelial tight junctions, which consequently leads to a chronic wound state and persistence of the factors underlying wound repair thereby contributing to the structural changes imposed by airway remodeling [31,32].

Intracellular signaling between BECs and HLF has been previously demonstrated in other models. Lama and colleagues demonstrated that murine fibroblasts grown alone in cell culture exhibited increased cellular proliferation compared to those grown in co-culture with murine AECs. Using cells derived from transgenic mice, these investigators additionally linked the down regulation of cells grown in co-culture to the COX-2 pathway and the production of the anti-proliferative signaling of PGE_2_ [33]. Hostettler and colleagues demonstrated a ~50% reduction in fibroblast proliferation following incubation of cultured HLF cells with conditioned media obtained from BECs [34]. Noteworthy, pre-incubation with a PGE_2_ inhibitor negated this effect. In the same series of experiments the authors demonstrated that adding TGFβ neutralizing antibodies to the conditioned media also blocked the inhibitory effect and concluded that TGFβ was likely involved in the regulation of the PGE_2_ axis. These studies highlight the important concept that is that crosstalk between the BECs and fibroblast cells may impact FMT and subsequent airway repair or remodeling. Additional studies have highlighted the effects of fibroblasts on AEC proliferation; however, our study was not designed to investigate this interaction [35,36].

Recently our group has reported different signaling profiles between healthy BECs and asthmatic BECs using our BEC-HLF co-culture model [11]. In those series of experiments, both healthy and asthmatic subject derived BEC were differentiated at an ALI and subsequently co-cultured with a common HLF cell line. Analysis of the media following 96 hours of co-culture revealed significantly greater amounts of TGFβ_2_ in the asthmatic BEC-HLF co-cultures. In contrast, prostaglandin E2 (PGE_2_) synthase gene expression was diminished in the asthmatic BEC-HLF co-cultures. These findings correlated with an increased expression of several ECM components by the HLF including collagen types I and III, hyaluronan, and fibronectin. Thus, these findings support a shift in the balance of signaling mediators toward a more pro-fibrotic milieu. The findings of the present study extend the these findings by demonstrating a diminished regulation of FMT in the asthmatic BEC-HLF co-cultures suggesting that the enhanced production of ECM components is related to an increased presence of myofibroblasts in the asthmatic BEC co-cultures. In addition, the use of TGFβ_2_ Mab in the present study further demonstrates the importance of the TGFβ_2_ signaling pathway in regulation of FMT. Thus, targeted disruption of the signaling that facilitates FMT may have direct implications for the regulation of ECM deposition in this model system as well as asthmatic airway remodeling and will be the focus of future investigations.

Our study design does have some inherent limitations. The population of subjects with asthma exhibited mild airflow obstruction, which is consistent with milder phenotypes of asthma. This finding may be independent of treatment or could alternatively be related to good adherence to controller medications given that 90% of the asthmatics studied were taking daily ICS at the time of enrollment. Since the cells used in the co-cultures were multiple passages beyond the initial sample collection it is unlikely that any medications being taken at the time of recruitment would still be present in the culture media or within the cells themselves. However, we can not exclude the possibility that the exposure to medications such as ICS, bronchodilators, or leukotriene receptor modulators might alter gene expression via epigenetic or other lasting genetic regulatory mechanisms as information on the occurrence of such processes in humans is lacking. If present, these effects would likely bias toward the null hypothesis and make differences in BEC regulation of FMT between the groups less apparent. Our asthmatic subjects had significantly lower FEV_1_/FVC ratios than the healthy subjects, indicating the presence of some degree of airflow obstruction despite a relatively mild asthma phenotype. An additional limitation to our cohort is that the number of males and females is unbalanced. With the relatively small number of subjects in each group, it is difficult to make meaningful comparisons between male and female subjects. Given the findings of the present study in conjunction with our previously reported findings, additional investigation of signaling pathways and the balance between pro-fibrotic and anti-fibrotic signaling pathways is warranted in future studies. Such investigation may lead to a better understanding of how FMT may contribute to the disordered wound repair and abnormal deposition of ECM seen in asthmatic airway remodeling. This is complicated by challenges involved in identifying a specific myofibroblast marker. The topic of which array of markers identify this cell type has been an area of debate in the pulmonary fibrosis literature for some time. Most investigators acknowledge that α-SMA is highly correlated with the myofibroblast phenotype; however, the specificity of markers is variable [17,26]. In the present study we chose α-SMA expression as our marker of FMT and evaluated its expression using multiple techniques. We chose to evaluate the expression of tropomyosin-I as an additional marker that could be evaluated by gene expression analysis. We observed that similar to α-SMA expression tropomyosin-I expression was increased following co-culture with asthmatic BEC compared to healthy BEC. Interestingly, we did not observe increased expression of tropomyosin-I in the HLF that were cultured alone suggesting that there may not be a 1:1 link between the expression of these two markers of FMT or that they are governed by different regulatory mechanisms. Further delineation of these mechanisms is beyond the scope of the present study, but will be an important consideration for future investigation.

Another limitation of this study is that our model is an *in vitro* model of cellular function; however, similar *in vivo* studies in healthy and asthmatic children would not be ethical or feasible. The findings from studies such as these should be verified in animal models of asthma. The latter may allow for more mechanistic studies to identify important signaling axes. Despite these limitations our study design does have several inherent strengths. We have used a common healthy donor HLF cell line for the experiments, which helps to isolate differences seen between the healthy and asthmatic groups to activity of the BECs. This limits the biologic variability of the HLFs; however, it is important to point out that our HLF are also human cells, which are subject to inherent genetic variability. Our experimental design highlights the differences between the healthy and asthmatic derived BECs, thus exploration of the effects of alterations within the HLF is not possible in the current study. Future studies to demonstrate reproducibility and explore alterations intrinsic to HLF, i.e. healthy vs. asthmatic derived HLF, will be needed. Furthermore, we are able to clinically characterize our population based on medical history, laboratory testing, and lung function, allowing for strict inclusion and exclusion criteria of the study subjects.

## Conclusions

In this study, we have demonstrated that HLF cells from a common healthy donor behave differently when co-cultured with BECs obtained from a healthy donor than when co-cultured with BECs obtained from an asthmatic donor, suggesting that intracellular signaling between the these two cell populations is important for the regulation of HLF phenotype. Our finding that HLFs co-cultured with BECs derived from asthmatic subjects display a measurably greater degree of FMT compared to those co-cultured with BECs derived from healthy subjects may explain the greater degree of subepithelial ECM deposition that has been described previously in biopsy specimens from asthmatics [9,10]. Furthermore, this finding is also consistent with our previous report of enhanced ECM deposition in similar co-culture experiments. Future studies aimed at the delineation of the signaling mechanisms underlying the intracellular crosstalk between BECs and HLF, particularly those underlying the differentiation of myofibroblasts, are warranted and may provide additional insight into the development of airway remodeling in childhood asthma and help identify new therapeutic targets to prevent remodeling and declines in lung function.
